# Supplementary figures and images for: Evidence for an oncogenic role of HOXC6 in human non-small cell lung cancer
Source: PeerJ. 2019 Apr 9;7:e6629. doi: 10.7717/peerj.6629 (PMC6461029; doi:10.7717/peerj.6629)

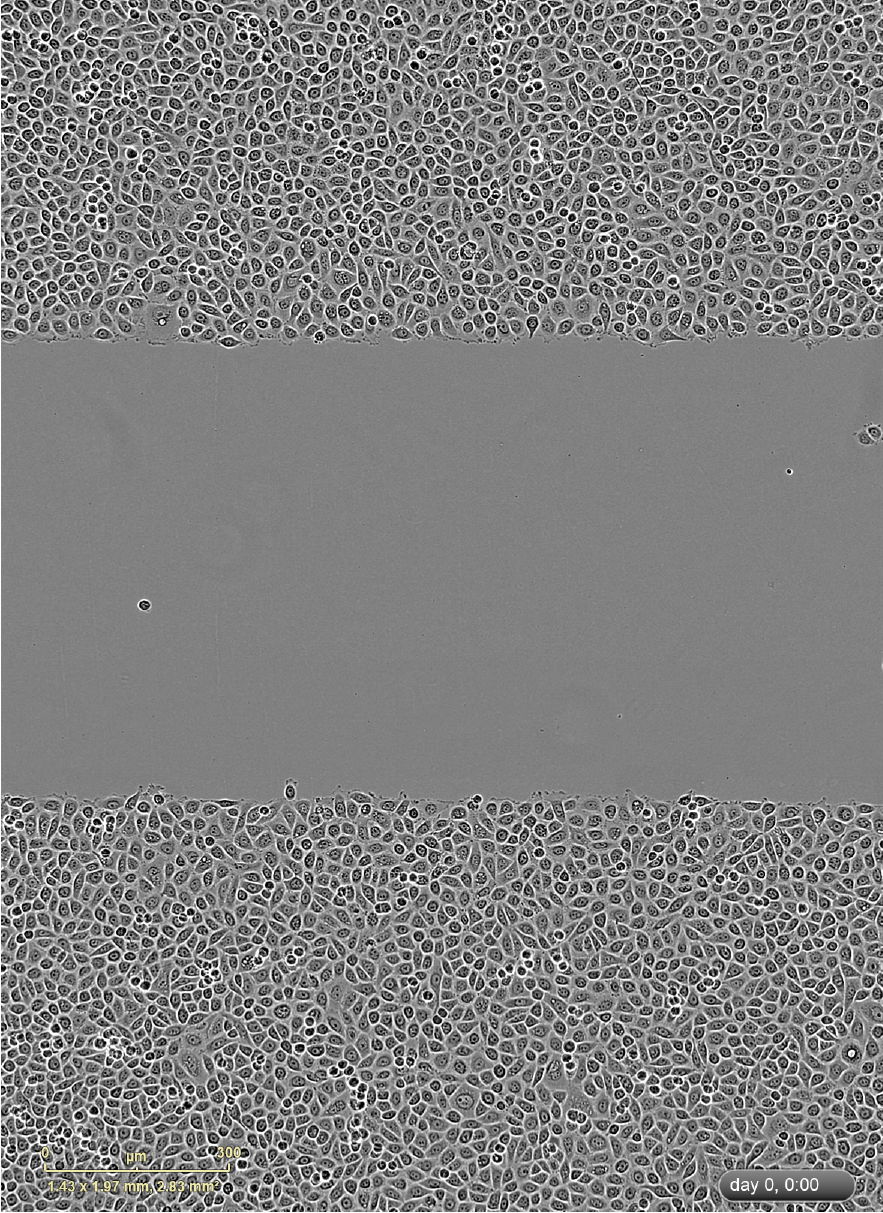

Supplement: Supplemental Information 1 [file peerj-07-6629-s001.zip › RAW DATA/Fig. 3 picture/2018-1-12-PC9-HH-hoxc6_E12_1_2018y01m12d_10h00m.png]

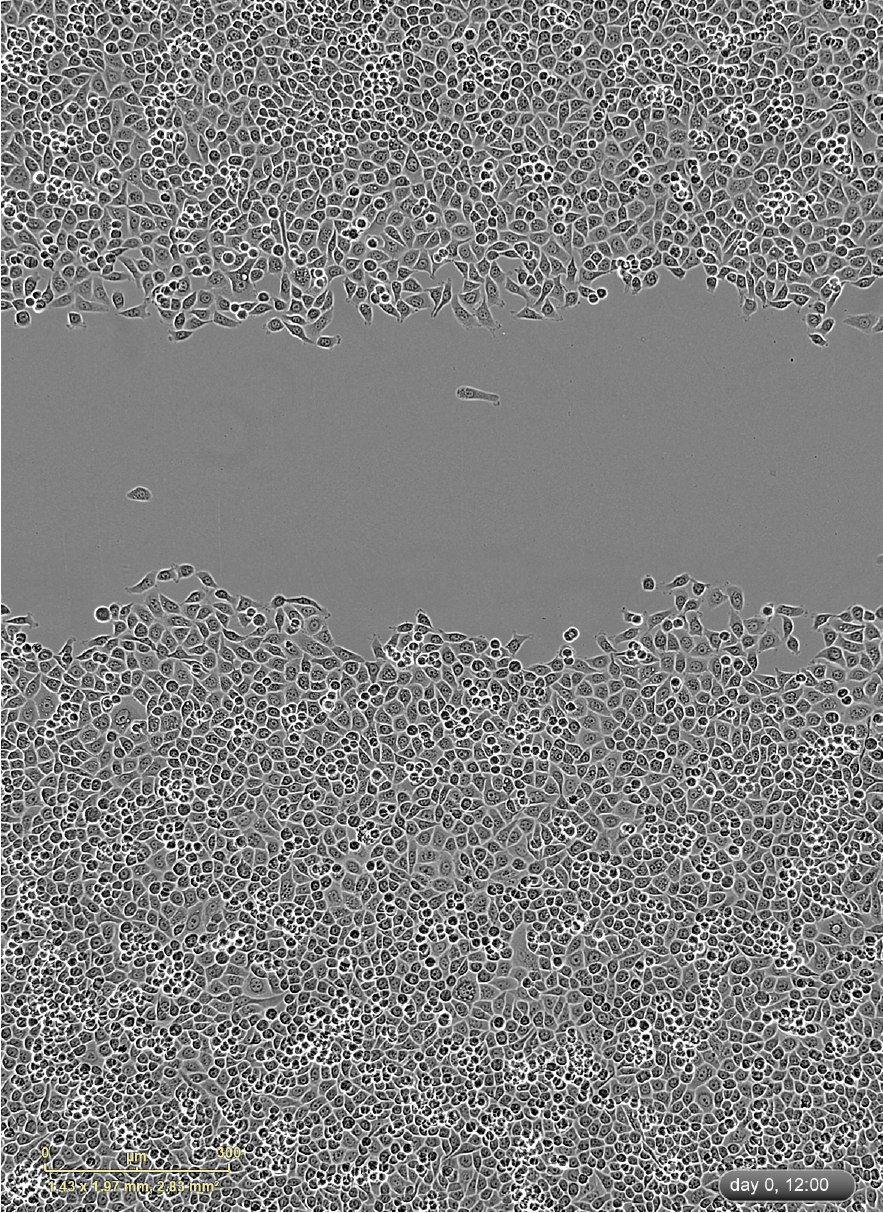

Supplement: Supplemental Information 1 [file peerj-07-6629-s001.zip › RAW DATA/Fig. 3 picture/2018-1-12-PC9-HH-hoxc6_E12_1_2018y01m12d_22h00m.png]

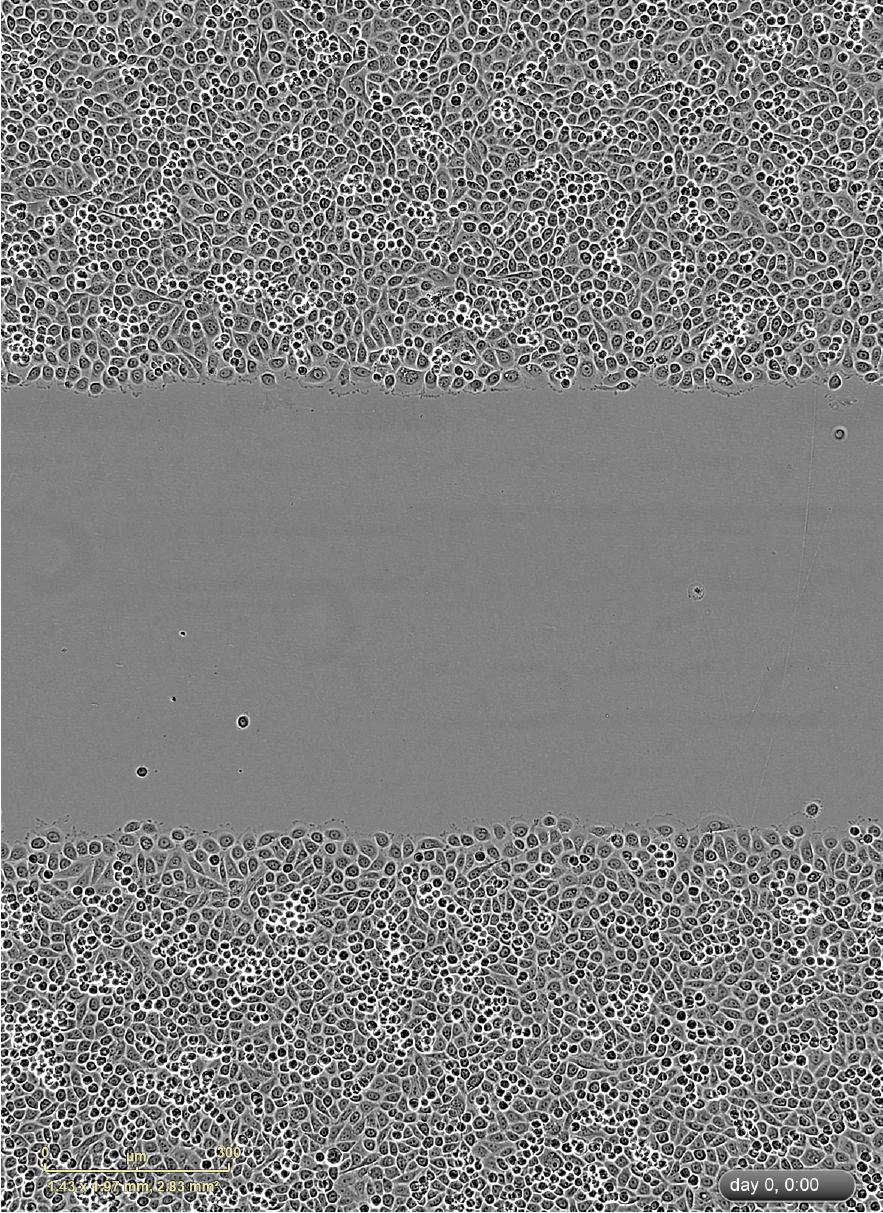

Supplement: Supplemental Information 1 [file peerj-07-6629-s001.zip › RAW DATA/Fig. 3 picture/2018-1-12-PC9-hh-neo-hoxc6_D4_1_2018y01m12d_10h00m.png]

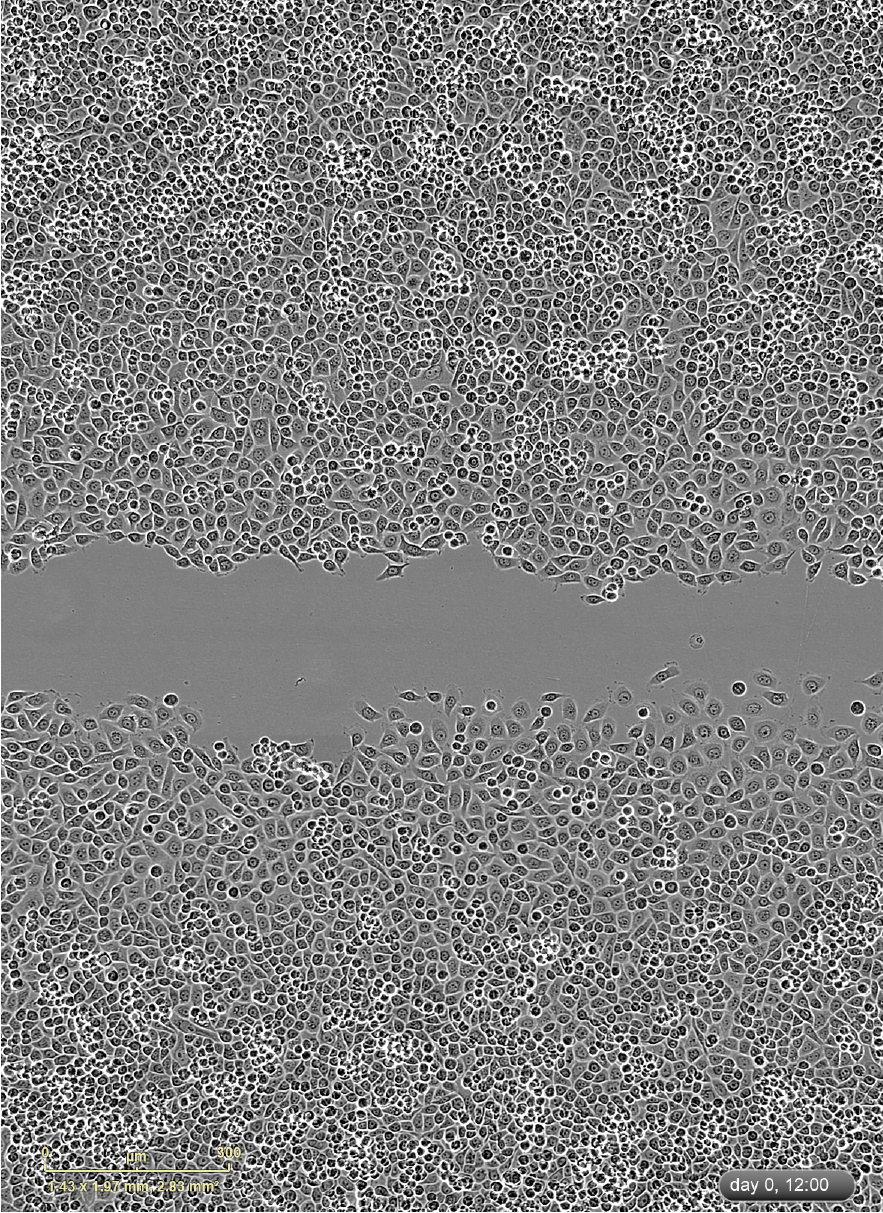

Supplement: Supplemental Information 1 [file peerj-07-6629-s001.zip › RAW DATA/Fig. 3 picture/2018-1-12-PC9-hh-neo-hoxc6_D4_1_2018y01m12d_22h00m.png]

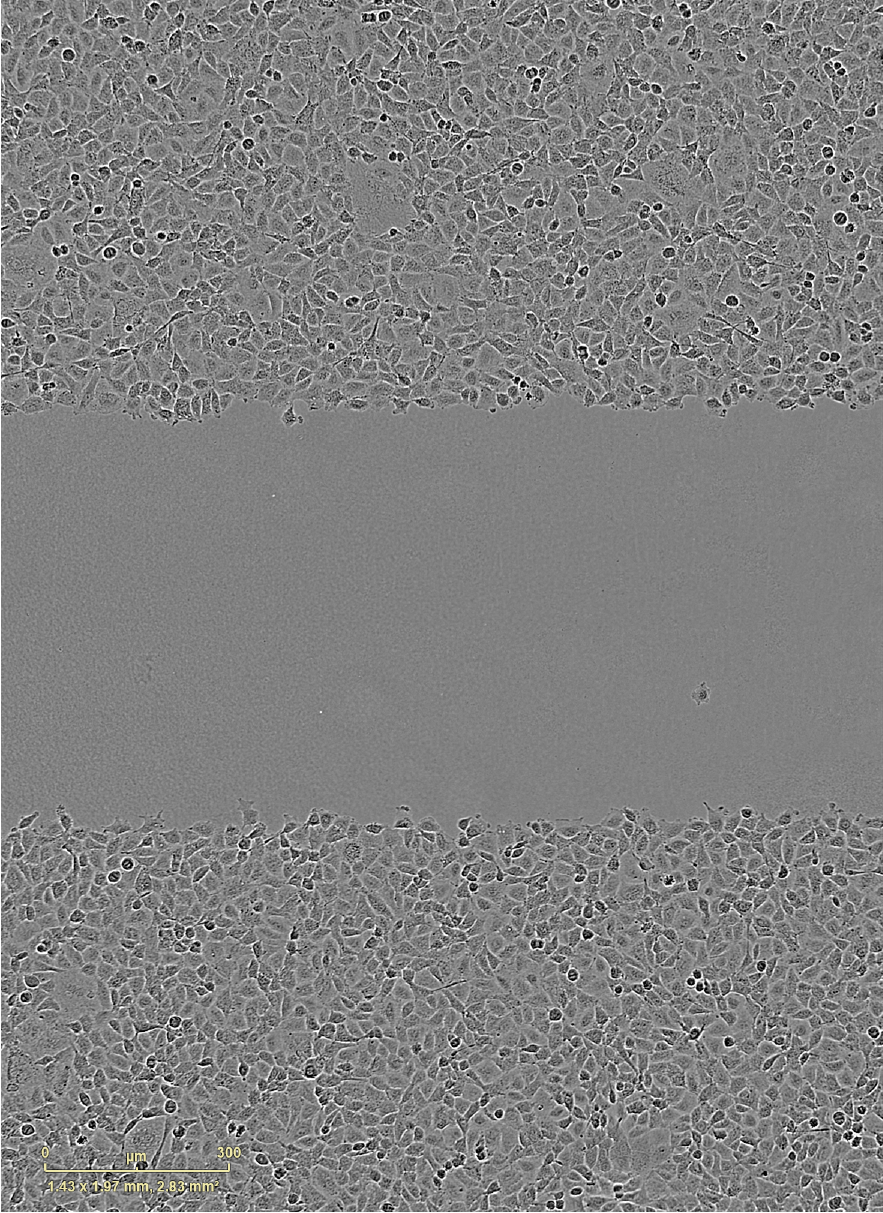

Supplement: Supplemental Information 1 [file peerj-07-6629-s001.zip › RAW DATA/Fig. 3 picture/2018-3-20-A549-neo-30%matrigel_D2_1_2018y03m20d_11h10m.png]

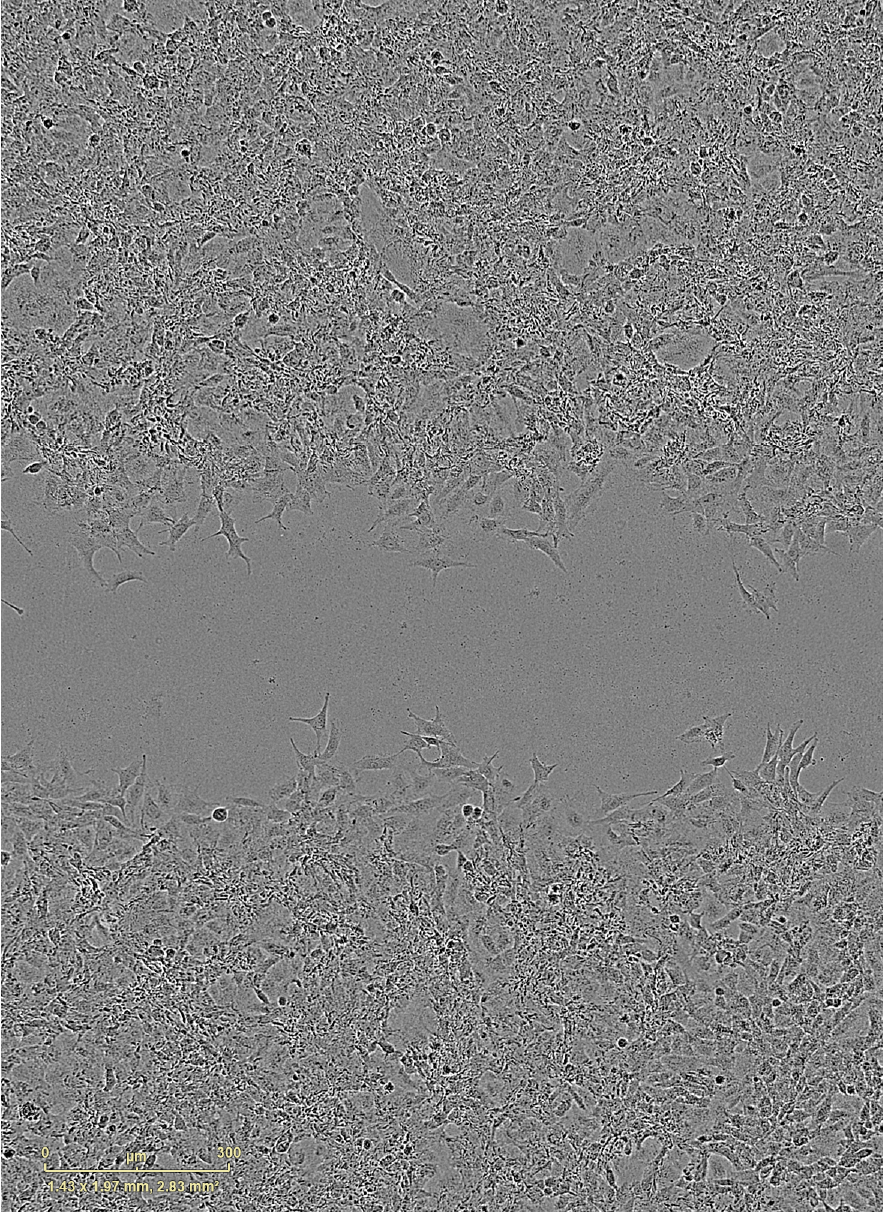

Supplement: Supplemental Information 1 [file peerj-07-6629-s001.zip › RAW DATA/Fig. 3 picture/2018-3-20-A549-neo-30%matrigel_D2_1_2018y03m21d_11h10m.png]

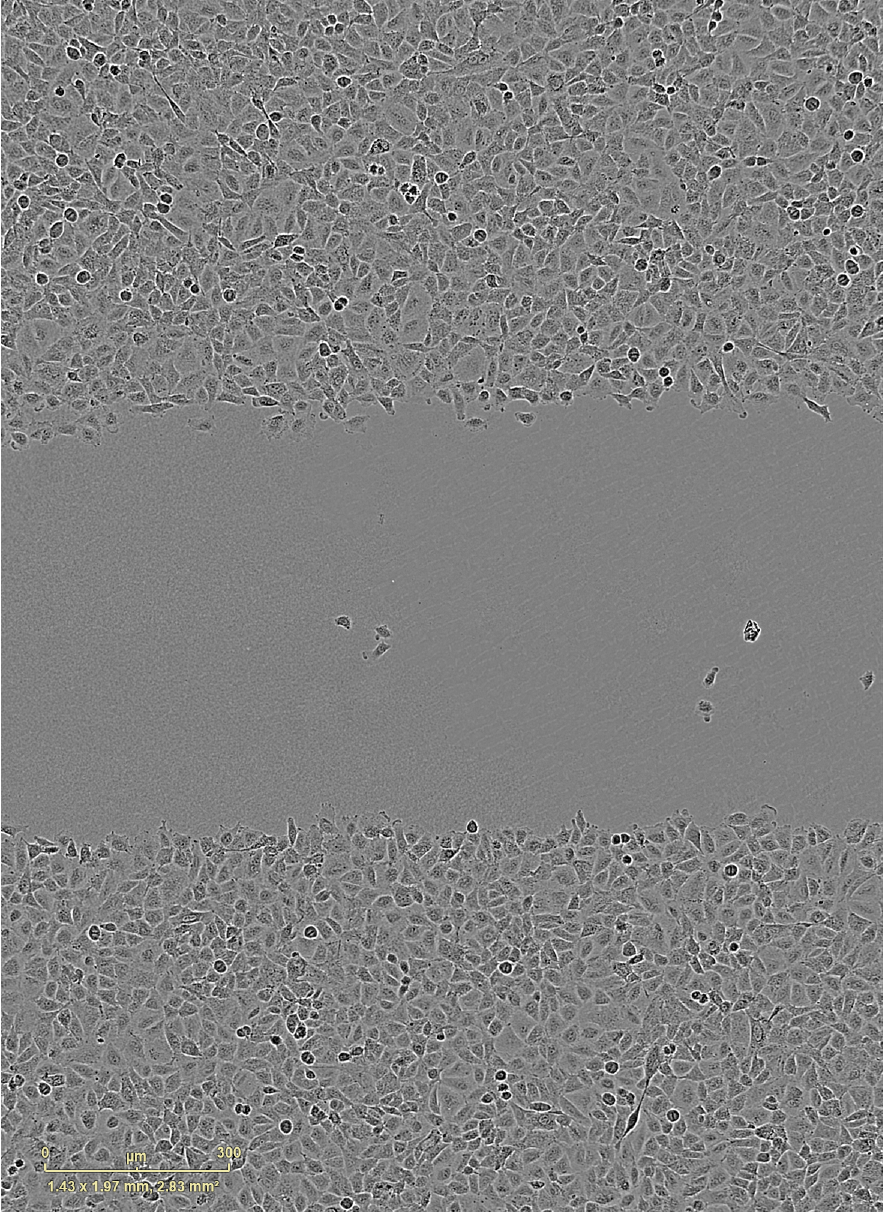

Supplement: Supplemental Information 1 [file peerj-07-6629-s001.zip › RAW DATA/Fig. 3 picture/2018-3-20-A549-neo-hoxc6-30%matrigel_A5_1_2018y03m20d_11h10m.png]

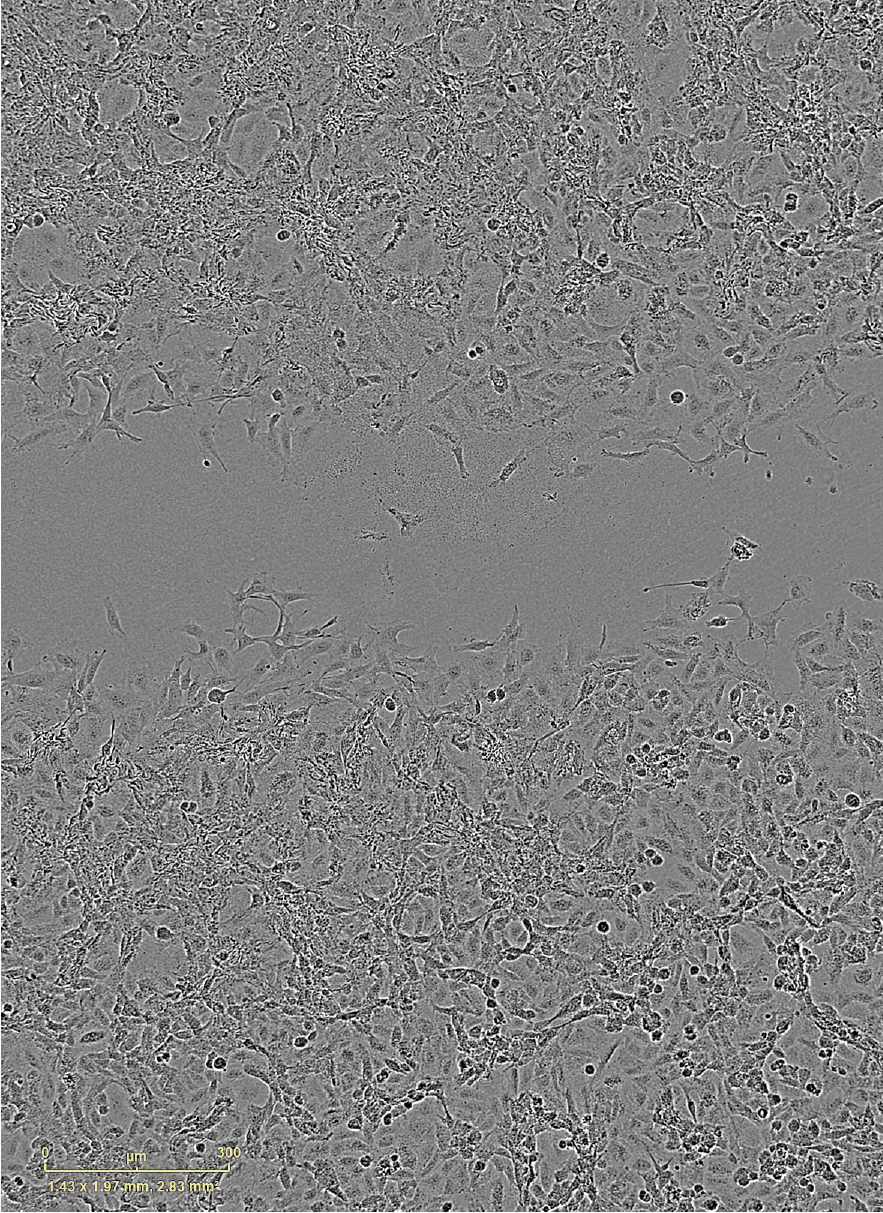

Supplement: Supplemental Information 1 [file peerj-07-6629-s001.zip › RAW DATA/Fig. 3 picture/2018-3-20-A549-neo-hoxc6-30%matrigel_A5_1_2018y03m21d_11h10m.png]

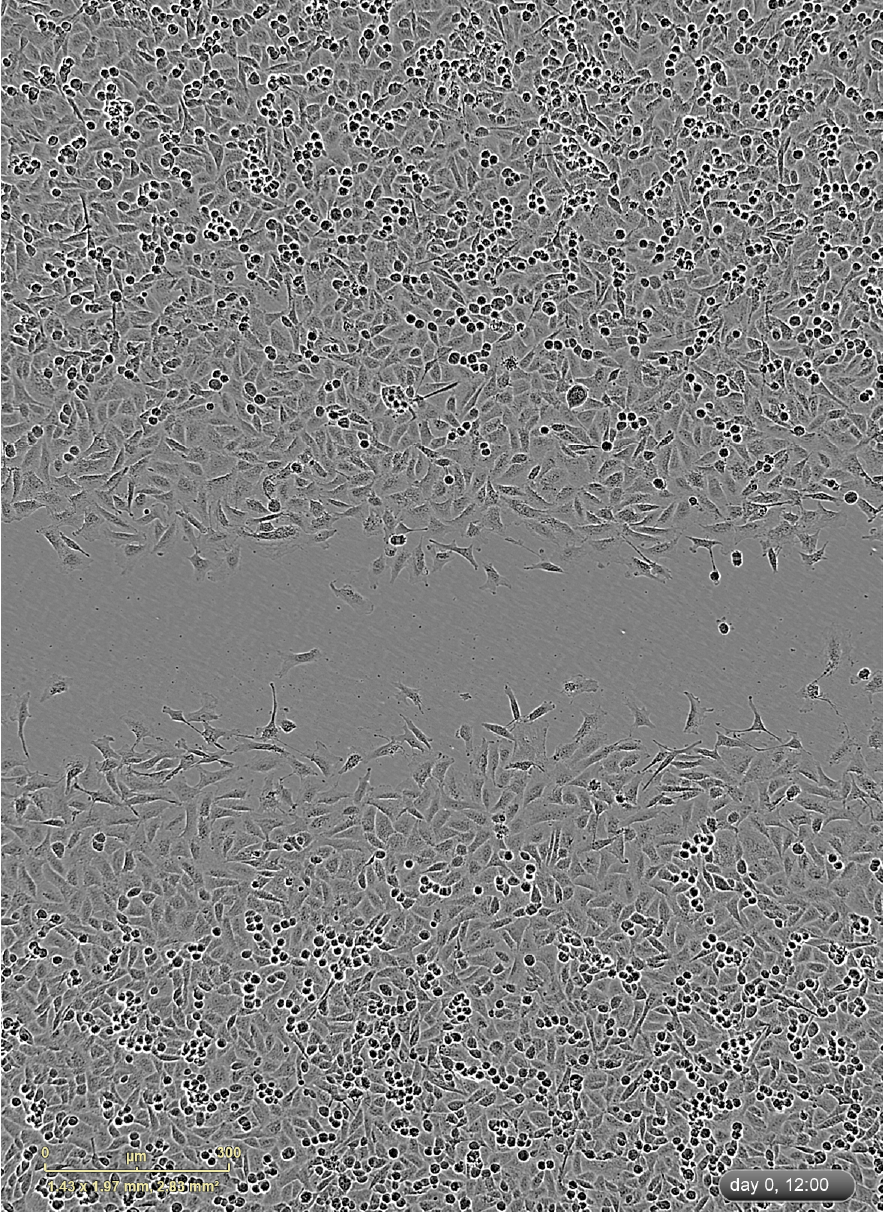

Supplement: Supplemental Information 1 [file peerj-07-6629-s001.zip › RAW DATA/Fig. 3 picture/2018-3-20-a549-HH-HOXC6_G5_1_2018y03m20d_23h10m.png]

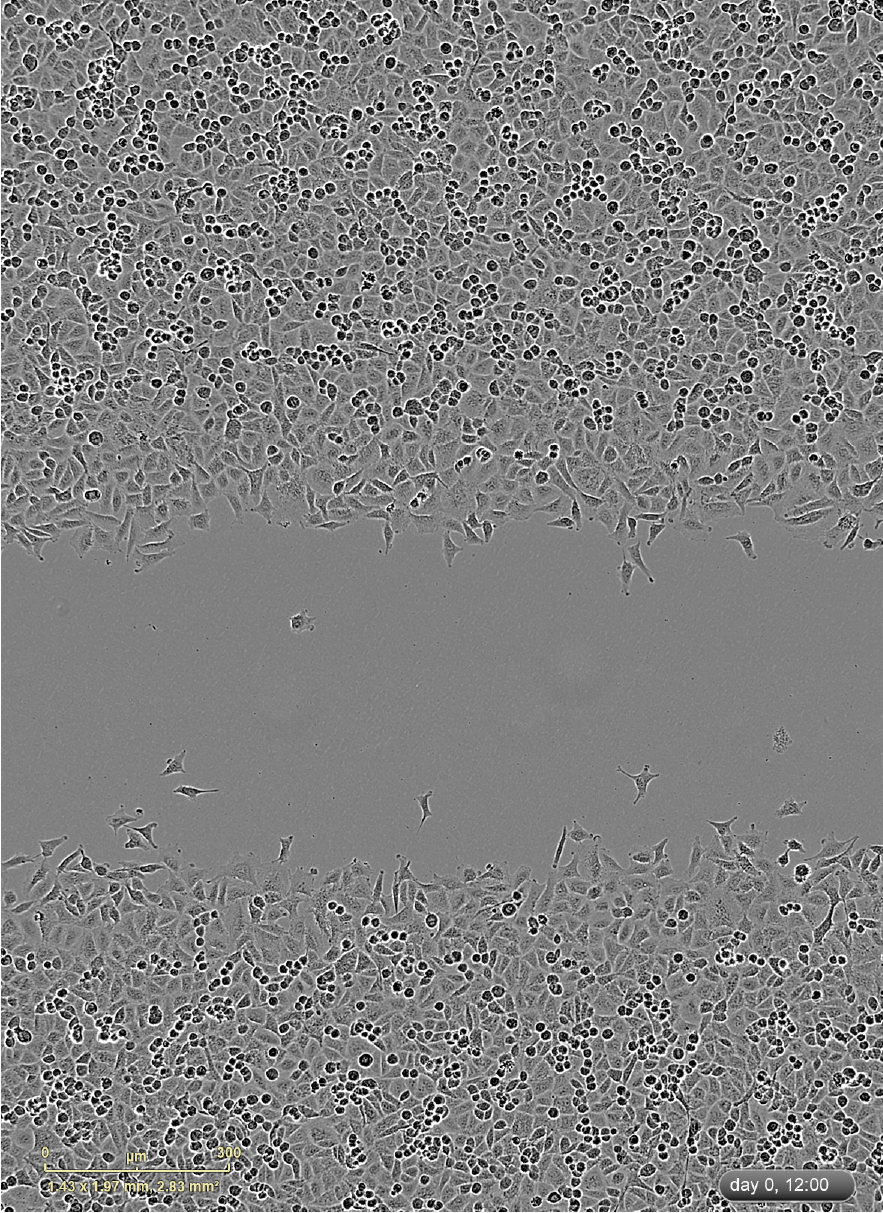

Supplement: Supplemental Information 1 [file peerj-07-6629-s001.zip › RAW DATA/Fig. 3 picture/2018-3-20-a549-HH-HOXC6_G5_1_2018y03m20d_23h10m1.png]

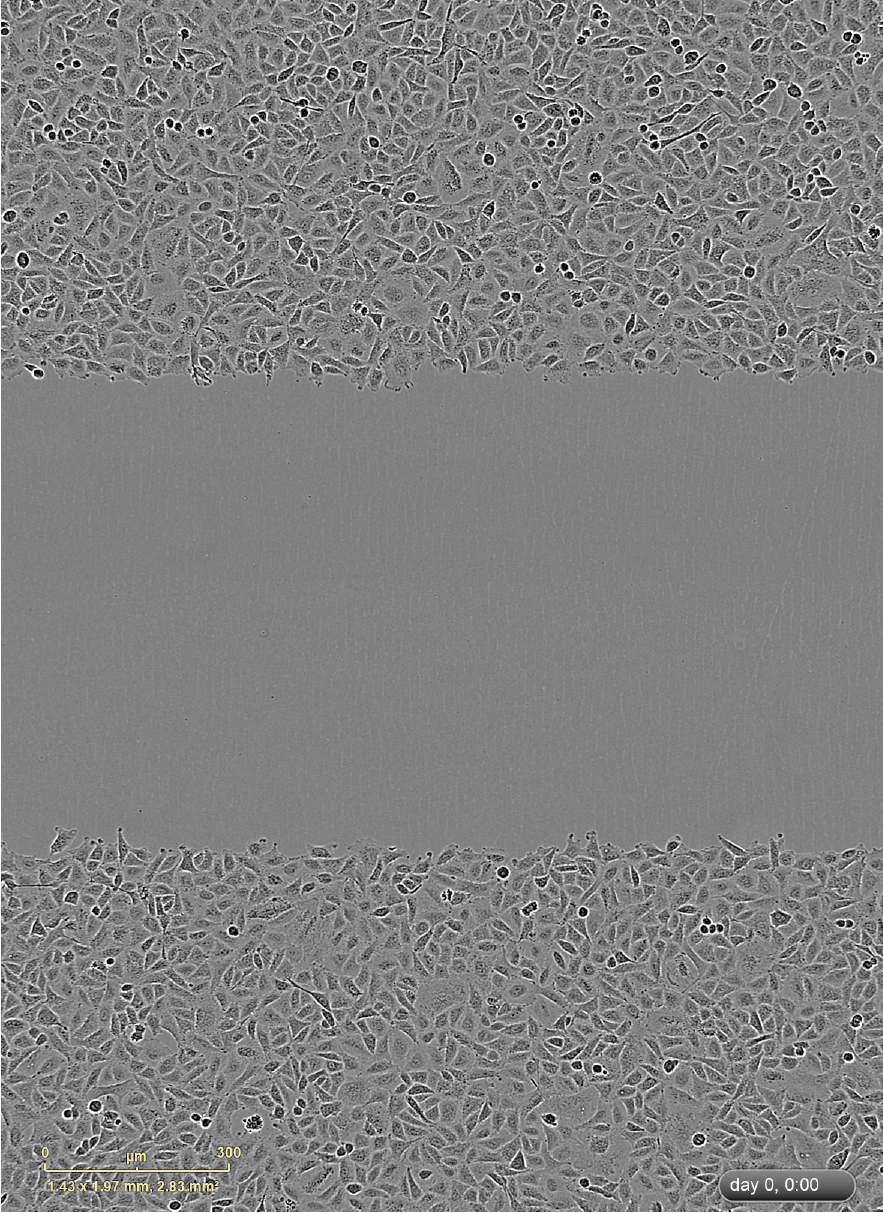

Supplement: Supplemental Information 1 [file peerj-07-6629-s001.zip › RAW DATA/Fig. 3 picture/2018-3-20-a549-HH-NEO_D1_1_2018y03m20d_11h10m.png]

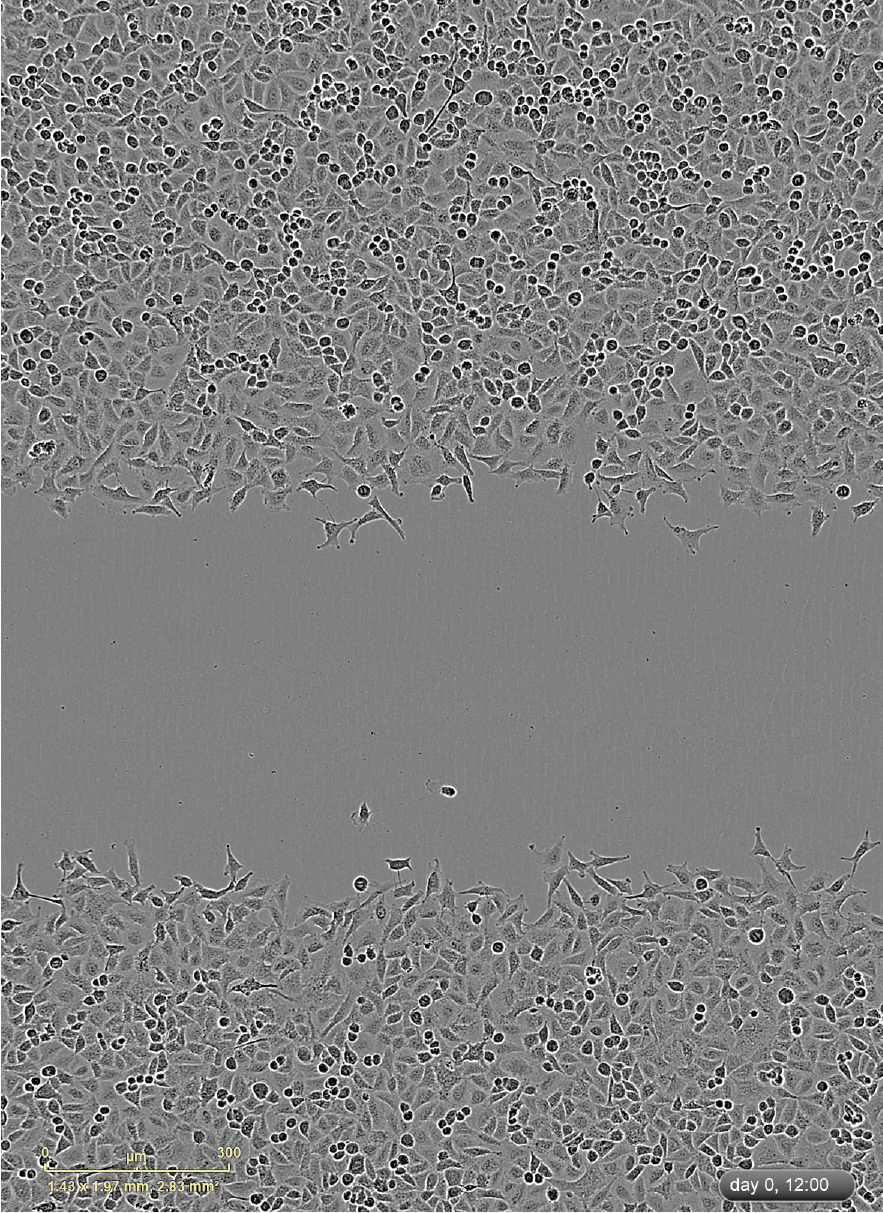

Supplement: Supplemental Information 1 [file peerj-07-6629-s001.zip › RAW DATA/Fig. 3 picture/2018-3-20-a549-HH-NEO_D1_1_2018y03m20d_23h10m.png]

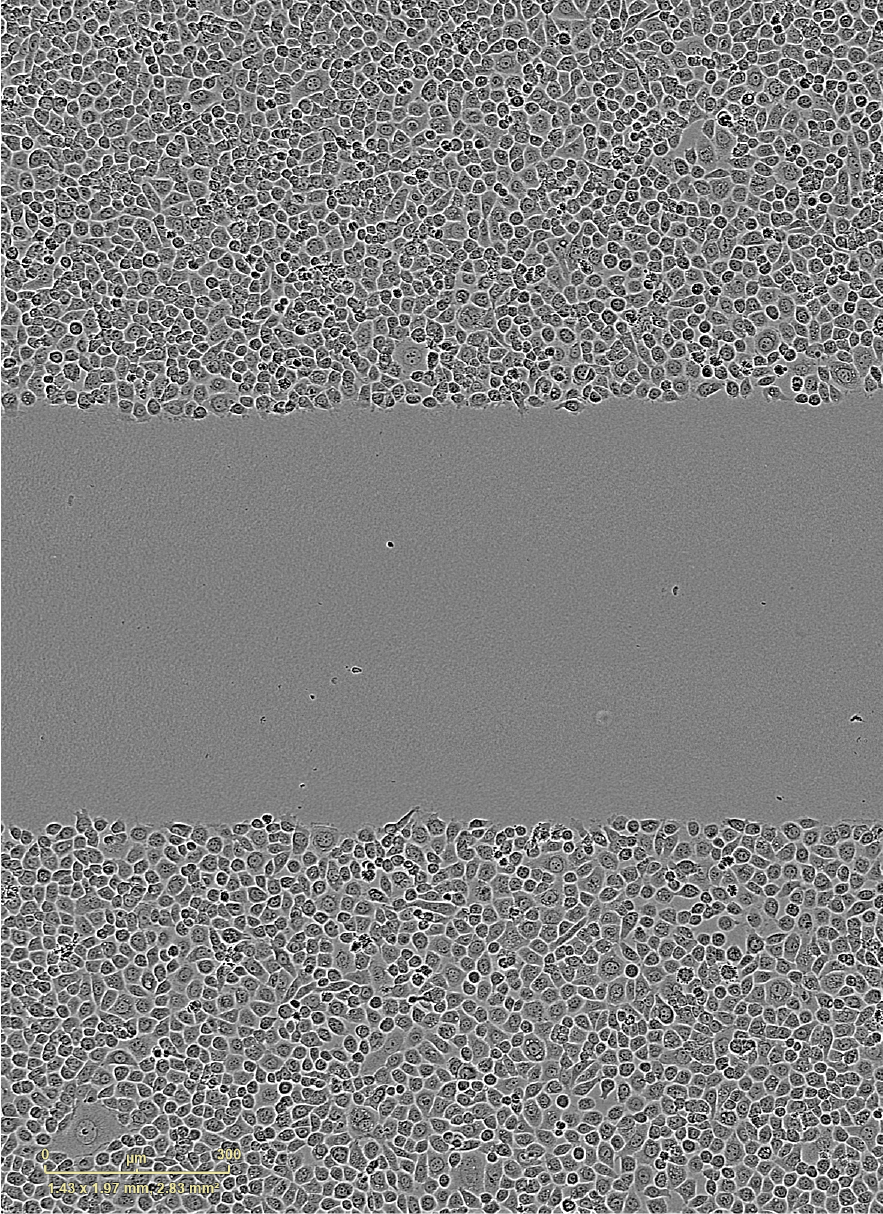

Supplement: Supplemental Information 1 [file peerj-07-6629-s001.zip › RAW DATA/Fig. 3 picture/2018-6-20-PC9-neo-30%matrigel_B2_1_2018y06m20d_11h15m.png]

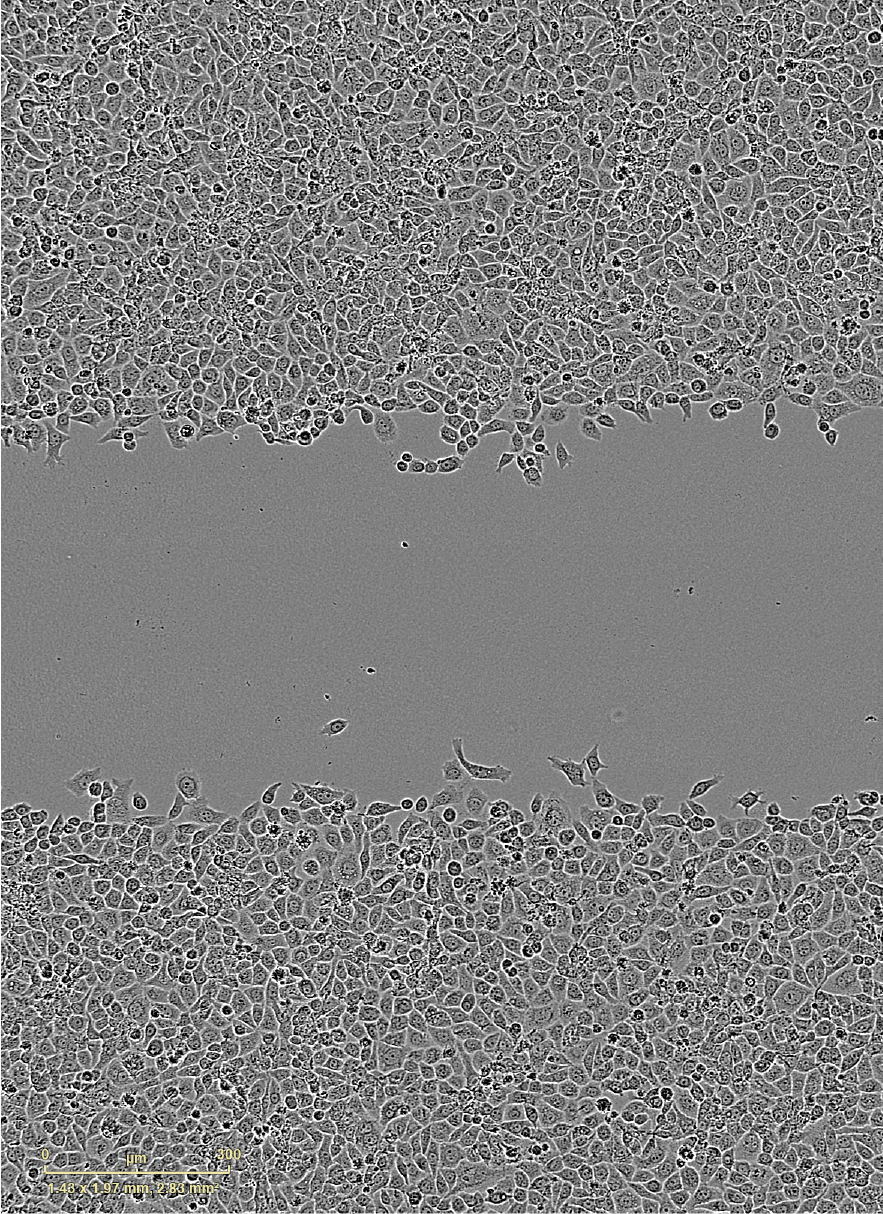

Supplement: Supplemental Information 1 [file peerj-07-6629-s001.zip › RAW DATA/Fig. 3 picture/2018-6-20-PC9-neo-30%matrigel_B2_1_2018y06m20d_23h15m.png]

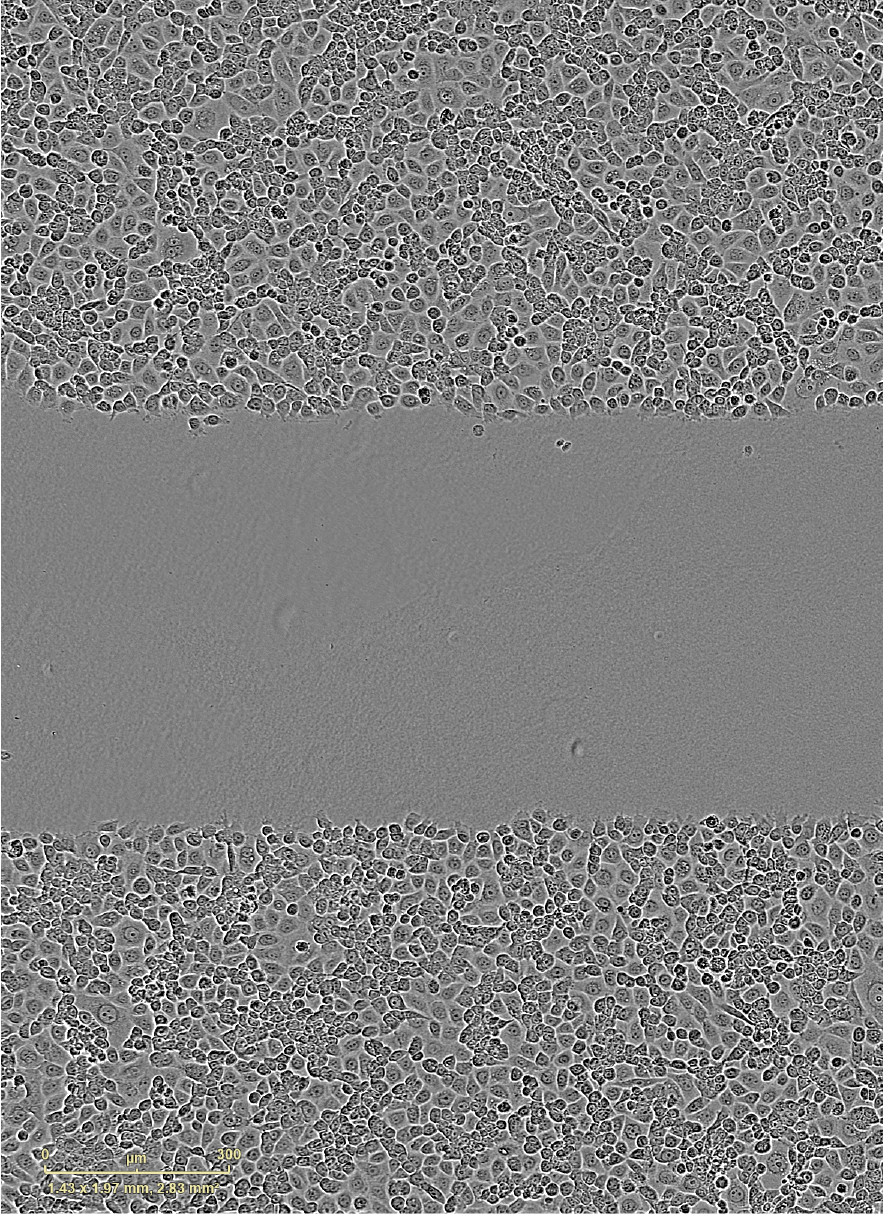

Supplement: Supplemental Information 1 [file peerj-07-6629-s001.zip › RAW DATA/Fig. 3 picture/2018-6-20-PC9-neo-hoxc6-30%matrigel_H10_1_2018y06m20d_11h15m.png]

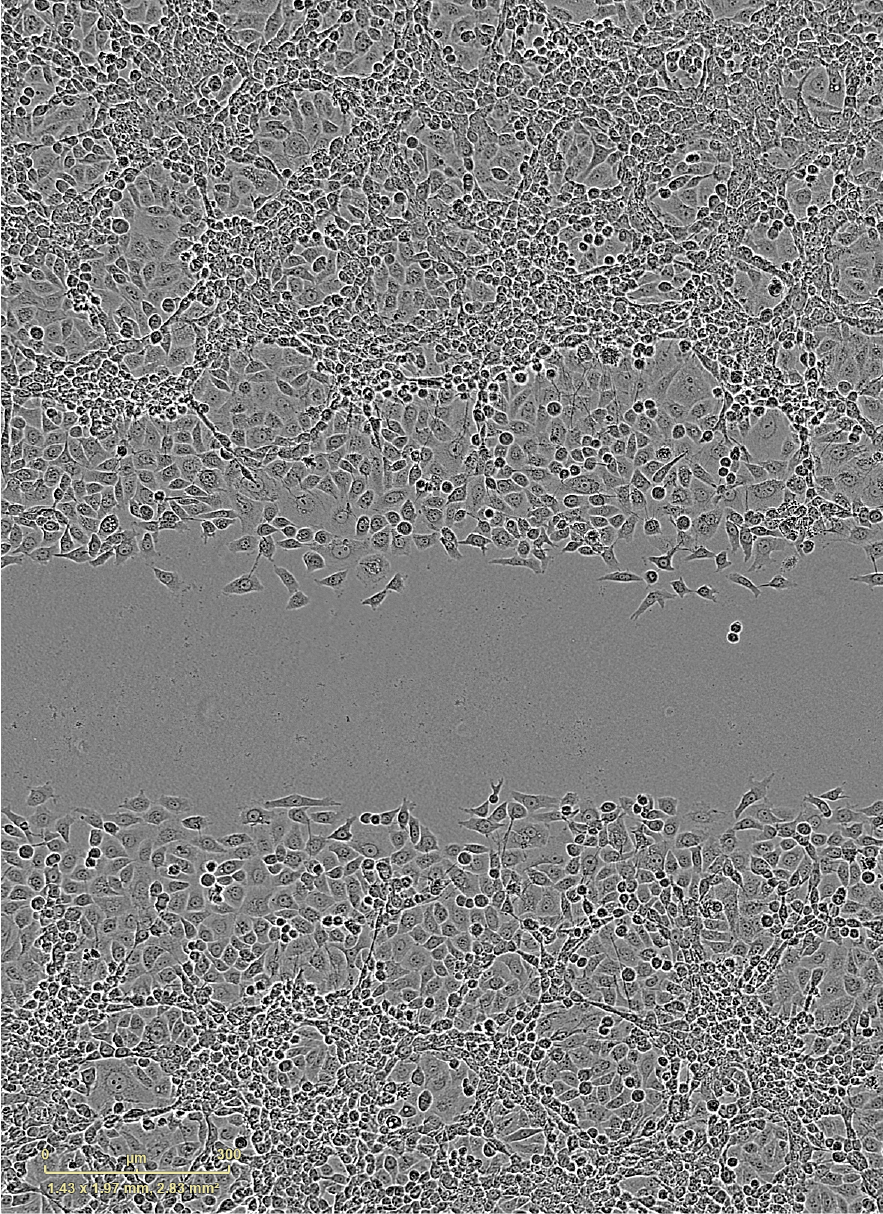

Supplement: Supplemental Information 1 [file peerj-07-6629-s001.zip › RAW DATA/Fig. 3 picture/2018-6-20-PC9-neo-hoxc6-30%matrigel_H10_1_2018y06m20d_23h15m.png]
